# “It’s like a safety net for when things go wrong”: key stakeholder and program user perspectives on a peer-led safe space program in Sydney, Australia

**DOI:** 10.1186/s12954-023-00854-2

**Published:** 2023-09-09

**Authors:** Phillip Wadds, Christopher M. Doran, Anthony Shakeshaft, Dam Anh Tran

**Affiliations:** 1 School of Law Society and Criminology, Faculty of Law and Justice, University of New South Wales, Sydney, NSW 2052 Australia; 2 Cluster for Resilience and Well-Being, School of Health, Medical and Applied Sciences, Central Queensland University, Rockhampton, Australia; 3 Centre for Indigenous Health, Faculty of Health and Behavioural Sciences, University of Queensland, St Lucia, Australia; 4 NHMRC Clinical Trials Centre, The University of Sydney, Sydney, Australia

**Keywords:** Alcohol, Illicit drugs, Alcohol-related harm, Harm reduction, Night-time economy, Entertainment precinct, Policing, Safety, Safe spaces, Peer-to-peer services

## Abstract

**Background:**

Safe Spaces are a harm reduction approach commonly utilised in nightlife and festival settings to address alcohol and other drug-related harms. Despite increasing use, there has been little independent evaluation of safe space programs. This study aimed to explore (1) program user satisfaction with and use of a safe space program implemented in Sydney, Australia (*The Take Kare Safe Space (TKSS*)), and (2) the strengths and weaknesses of TKSS from the perspective of key stakeholders.

**Methods:**

Semi-structured, in-depth, interviews lasting between 30 min to 1 h were conducted with 38 key program stakeholders, including staff from police (*n* = 4), ambulance (*n* = 4), a local hospital accident and emergency room (*n* = 4), local council (*n* = 2), city ‘rangers’ (*n* = 2), the TKSS program (*n* = 4), licensed venues and other nightlife service providers (*n* = 4), and program users (*n* = 14). Purposive sampling was used to identify key stakeholders to participate in interviews.

**Results:**

Stakeholders stated that the TKSS program had a number of core benefits, including that it filled a service gap in nightlife settings; improved the efficiency and effectiveness of emergency services and other stakeholders operating in nightlife precincts; provided welfare services through proactive and non-judgmental interventions; and facilitated a means to de-escalate conflict without engaging police. Perceived weaknesses of the program included a lack of public awareness about the program; staff and volunteer levels; and misunderstandings regarding the scope and function of the TKSS program by some stakeholders.

**Conclusion:**

This study demonstrates the complex relationships that exist around the delivery of harm reduction in nightlife settings. In particular, it highlights the relative lack of servicing of public nightlife settings and the value of safe spaces/peer-to-peer safety ambassador programs in linking up care and filling this service gap. Further, it documents the extended benefit across key stakeholder groups of delivering proactive and non-judgemental harm reduction services and, in doing so, provides critical evidence around their efficacy in reducing AOD-related harms in the night-time economy.

## Introduction

The ‘night-time economy’ (NTE) is an important part of contemporary social and economic life, referring to the mix of business, leisure, social and cultural activities that take place in the evening, generally from 6 pm onwards [1–5]. In the last two decades, research into the NTE has demonstrated that particular forms of nightlife have become synonymous with monocultures of ‘determined’ alcohol [2, 6–8] and drug-based [9, 10] intoxication that often result in disorder and acts of violence [2, 7, 8, 11–20]. Alcohol and other drug (AOD) use has been shown to increase the likelihood and extent of aggressive, violent and risk-taking behaviour [18, 21], to be a major factor in around a third of violent crime [22], a significant contributor to homicides [23], assaults [20, 24], sexual violence in commercial leisure settings [25–28], and 34% of all road fatalities [29]. These harms have significant and lasting effects across the community on an individual, social, and economic level.

Licensed entertainment precincts and venues are high risk settings for AOD-related violence, with a significant proportion of assaults occurring in or within close proximity to these locations [30–32]. Links are also consistently found between licensed venue density and violence [33–36]. In New South Wales, Australia, where this project was undertaken, evidence confirms that most alcohol-related violence (84%) occurs at night between 6 pm and 6am, with alcohol linked to almost half (47%) of all assaults on the weekend [20]. Harms experienced in and around licensed settings can be exacerbated by poor regulatory controls and punitive or aggressive interventions, including non-compliance with responsible service of alcohol standards [37], confrontational entry and ejection practices [37], poor venue management [38], lax surveillance [38] and/or aggressive policing tactics [2, 27, 39], lack of transport options [24, 37], and inappropriate bureaucratic controls and legislation [2, 24, 38].

Around the globe, there have been growing number of services, programs, campaigns and interventions introduced (and evaluated) in an attempt to prevent and/or reduce the impact of AOD consumption and related violence in the NTE [40–47]. These interventions target a range of ‘risk factors’ for AOD-related violence and harm, including by reducing alcohol availability through trading restrictions or one way door policies [48–53], addressing irresponsible/ non-compliant service of alcohol practices [40, 54–57], and providing better late night public transport options and support for night-time revellers [58–60]. Regarding the latter, one such approach is the establishment of ‘safe spaces’ or safe night precincts in night-time entertainment settings. Safe spaces are HR services often run in collaboration with health, community, emergency or welfare services to improve safety and amenity in public spaces by providing a combination of medical assessment, first-aid, counselling or support, hydration, supervised recovery, and/ or practical supports to individuals who are often AOD affected [61]. Common in the UK [61], and largely run via church outreach programs (often referred to as ‘street pastors’), many nightlife safe space services now operate in Australia [62–64]. Safe space programs are also often run at music festival events around the world [65–67]. Despite increasing implementation, there has been little rigorous independent evaluation of their impacts. While street pastor programs have received considerable academic attention, most of this has focused on the relationship between Christian outreach, moral governance and the night-time economy, rather than specifically analysing their impact on the prevention of harms that occur in nightlife settings (see, for example, [68–70]). Further, the few peer-reviewed evaluations published about safe space programs have all come from Australia bar one (which examined outcomes of the Kosmicare psychedelic harm reduction service at Boomtown Music Festival in Portugal [66]), and only four have reported on specific outcomes driven by programs (with another reporting on factors associated with engagement with a safe space service [62]). These studies have found mixed results around the efficacy of safe space programs in reducing AOD related harms. A study from Cairns, in North Queensland, Australia, for example, found that serious assaults during high-alcohol hours significantly declined after the introduction of a safe space service with a one-month lagged impact (*B* =  − 1.66, 95% confidence interval − 3.02, − 0.30) [71]. Another study by the authors of this paper reported on an economic evaluation and found that each dollar invested into a safe space program returned an estimated $2.67 in community benefit, including through reduced alcohol and drug related harm [64]. A third study from Melbourne, Australia examined a “shelter and van” service and found that while the service was well utilised and valued by nightlife revellers, there was no significant association between service provision and reduced alcohol-related hospital emergency department presentations or police recorded crime data [62]. The study that analysed outcomes of the Kosmicare psychedelic-specific harm reduction/ safe space service found statistically significant difference (*p* < 0.05) in crisis resolution following pre-post mental state evaluation [66]. Finally, a UK-based study which evaluated a Drinkaware program which included the use of roving ambassadors in nightlife settings in two unnamed cities showed an inconclusive effect of the initiative in the test venue in City A and a negative effect on assault and sexual assault at the test venues in City B [72]. All of these evaluations bar that conducted by Ward and colleagues [62] have relied heavily on quantitative data and analysis to speak to program impact. While important, this data tells only one part of the story when it comes to the delivery of safe spaces, with little to no consideration of the qualitative benefits and complex role that safe space/roving peer ambassador services can play in terms of the broader harm reduction/nightlife ecosystem they operate within. Critically, no research has documented the strengths and weaknesses of such programs as viewed by diverse nightlife stakeholder groups and policy actors. This data is critical in providing deeper insight into a range of less tangible outcomes and benefits of such initiatives, insights often overlooked in purely quantitative studies. In order to better inform policy makers, harm reduction practitioners and others working in nightlife settings, it is essential that empirical evidence for the effectiveness of interventions aimed at reducing alcohol-related harm is available and as comprehensive as possible.

To add to this limited knowledge base and provide a broader range of evidence into the role and benefits of safe space programs, this paper examines key stakeholder perspectives about the Take Kare Safe Space (TKSS) which operated in Sydney between 2014 and 2022.

## Background and context

The TKSS was a HR program implemented in Sydney in December 2014 with the aim of reducing AOD-related violence and crime by providing a place where vulnerable young people could access safety and support. The TKSS program provided safe spaces year-round from 10 pm to 4 am on Friday and Saturday nights in three areas of Sydney (Town Hall, Kings Cross and Darling Harbor) with vibrant NTEs. Each site of the TKSS program was staffed by multiple (usually between 1–3) groups of 3–4 team members, called Ambassadors, including a paid team leader and volunteers. Volunteers were drawn from the general public and student recruitment pathways from established relationships between the program and local universities (mainly paramedicine and social work students). These teams patrolled designated precincts to provide AOD-affected and other vulnerable people in unsafe situations with practical on-the-spot assistance, particularly in situations of “risk” (including high-level intoxication, conflict etc.). A static safe space was also provided. Run by team members, the static ‘space’ (a marquee with comfortable chairs) served as the ‘base’ from which Ambassadors patrolled and provided those engaging with the service a place to rest, receive first aid and hydration, charge mobile phones, find transport home, help connecting with friends or family, and general assistance. Integral to the TKSS program was its connection to, and interaction with, other agencies including City Rangers, licensed premises, venue security, police, closed circuit television, and transport staff. While initially starting as a collaboration between the Thomas Kelly Youth Foundation and service delivery partners the Salvation Army (December 2014 to June 2016) and St Johns Ambulance (July 2016 to June 2017), the TKSS moved to a non-affiliated, in-house delivery model in July 2017 until it ceased operations at the end of July 2022. All staff and volunteers were inducted into the program which involves basic first aid training and medical escalation protocol and de-escalation techniques.

## Methods

### Design

A mixed method, comprehensive evaluation study was conducted involving quantitative, qualitative and economic data collection and analyses. This paper reports on one part of this larger study, the qualitative interviews conducted with program users and stakeholders.

### Sample and recruitment

Semi-structured interviews were conducted with 38 program stakeholders, including staff from NSW Police (*n* = 4), NSW Ambulance (*n* = 4), St Vincent’s Accident and Emergency (A&E) staff (*n* = 4), City of Sydney Council (*n* = 2), the Darling Harbour Foreshore Authority (‘Rangers’) (*n* = 2), St John Ambulance (NSW) / the TKSS program (*n* = 4), licensed venues and other nightlife service providers (*n* = 4), and with program users (*n* = 14). Interview participants were recruited via two different methods. Interviews were across 2018 and 2019. For program users of TKSS, recruitment was completed via both a survey that was also conducted as part of the broader program evaluation (where participants interested in participating in an interview added their contact details to their survey for follow-up by the research team) and through direct advertisement with patrons using the service at night (via signage at the static site locations with QR codes attached to provide more information to potential participants). For stakeholders, targeted/purposive sampling and recruitment strategies were used. In most cases, including with police, A&E and ambulance staff, recruitment was supported by the research officers who had engaged with project staff during the required ethics approval processes for each organisation. Other stakeholders were recruited via direct email with known organisations. For key stakeholder groups, the final sample included key personnel working within these organisations who had operational knowledge of the TKSS program which accounts for the smaller number of participants for each stakeholder group. For program users, the final sample was determined through an iterative and reflexive design approach whereby the completed interview were routinely reviewed so the research team could assess the data and establish when data saturation had occurred.

### Data collection

Interviews followed a semi-structured interview guide that was developed via an iterative process utilising a review of the literature and input from an expert advisory group including experts from a peer-to-peer harm reduction service, emergency room medical and ambulance staff, the NSW Department of Justice, NSW Police as well as nightlife and public safety specialists from local council. Interviews with program users covered the following topics and themes: the nature of contact with the TKSS program; the general view towards the TKSS program; the perceived strengths and weaknesses of the program, areas for improvement. Beyond the above themes, stakeholders were asked about how the program had impacted their collaboration with other agencies operating at night and if the program had been of benefit to them in the execution of their work. Interviews were conducted both in-person and via telephone depending on participant location and preference. No costs were incurred by any participants, so no reimbursement was provided, and all participation was completely voluntary and audio-recorded with consent. The project received ethical review and clearance by UNSW HREC (HC17509), as well as from NSW Health and NSW Police.

### Analysis

Interviews were transcribed by a professional transcription service and subjected to thematic analysis by the lead author which identified and explored salient themes within and across the data set [73]. Analysis followed Braun and Clarke’s six step iterative process, involving data familiarisation, generating codes, generating themes based on salient codes, reviewing themes, naming themes and locating exemplars [73]. This work, including all coding and identification of themes was conducted by the lead author and cross-referenced with other members of the research team using NVivo. In the generation of themes, particular attention was paid to key points of repetition and divergence or difference, particularly across stakeholder groups.

## Results

### Identified strengths of TKSS

Identified strengths of the TKSS program were various and consistently shared across stakeholders, serving to reinforce the validity of findings. The most salient strengths identified across the interview dataset were: (1) the role of TKSS in filling a critical service gap in nightlife settings; (2) the benefits of providing early, proactive, non-judgmental interventions and de-escalating conflict; and (3) improving efficiency and effectiveness of emergency services and other stakeholders operating in nightlife settings.

1. *TKSS filled a critical service gap in nightlife settings.* All stakeholder groups interviewed, including program users, regarded the TKSS program as beneficial and thought it filled a long-existing gap in service provision in the *public* domain in the NTE. The TKSS program was said to offer a unique addition to the services and facilities already offered by established stakeholders such as licensees, police, and emergency health services, and contributed to the provision of a more complete suite of resources to manage Sydney nightlife. Key to this was the gap TKSS filled relating to welfare provision for vulnerable revellers in the city at night. Multiple interviewees reported a lack of appropriate care for those in public spaces who were heavily intoxicated. Stakeholders reported that overservice or irresponsible service of alcohol practices and/or pre-drinking by patrons prior to arrival in the city often resulted in heavily intoxicated patrons being ejected/refused entry from licensed venues. These practices create a service need for HR in the public domain of nightlife settings. Until the introduction of the TKSS program, multiple participants highlighted that appropriate care and supports had not been available to assist those in such situations, often resulting in ambulance or police interventions which were inappropriate, unnecessary, and often resulted in greater risk of harm (particularly relating to police intervention). Here, the introduction of the TKSS program was said to have provided a crucial harm reduction service, and stakeholders identified the ability of the TKSS program to recognise, access, engage and support vulnerable individuals as its primary function and greatest value-generating activity.

*‘For me, the program fills a gap, and that gap is: you don't need the police, you don't need an ambulance, you just need someone to help you if you're drug, alcohol affected or whatever’ (City of Sydney Staff #2)*

*‘Security and the venues don’t care. Security kick people out when they can barely stand up and just expect them to get home safely. They don’t think about the fact that they are splitting up your group, or that your phone is dead, or that you are too drunk to do anything but pass out in a corner somewhere…I guess we are just lucky that there is a service out there now that can look after you when this happens because I’ve been there and I know heaps of mates who have too’ (TKSS Program User #1)*

Many program users highlighted the reassurance provided by knowing the TKSS program was operating in the city at night providing management and surveillance of public space. Women highlighted that knowing roving Ambassadors were operating around sites of potential risk made them feel safer and provided a ‘beacon of hope’ and ‘a safety net’ if things went wrong.

*‘It just gives me kind of peace of mind that there are people out there for that because, obviously, the city is the kind of place people get intoxicated pretty much every night and there are, obviously, a lot of people who do drugs as well and it is very dangerous.’ (TKSS Program User #4)*

*‘It makes me feel safer just in general knowing that they're there and also, I think it’s nicer because, like, obviously, they're helping people, but they're also getting maybe people who are intoxicated or have drugs in their system get help, so they don’t then, you know, conflict with other people.’ (TKSS Program User #5)*

*‘It’s like a safety net for when things go wrong, they are there for you when you really need it’. (TKSS Program User #12)*

2. *The provision of early, proactive, non-judgmental interventions, and de-escalating conflict.* There was universal acknowledgement among TKSS program users and stakeholders that a key strength of the program was the provision of a non-judgmental and non-confrontational service. Program users highlighted a willingness to speak to TKSS staff in situations where they would usually be reluctant to divulge information that could see them “get in trouble”, particularly in instances where they were AOD-affected. Ambassadors were perceived as* “*very approachable” and “easy to talk to” and there was a clear preference among those interviewed to deal with peer groups who were less likely to judge them negatively, and who were able to help them feel at ease during often distressing and potentially dangerous situations.

*‘I felt like I could open up to them and kind of be like, “Yeah, I’m not feeling too good,” or, “Yeah, I’m feeling this,” or, “I’m feeling really anxious,” and they were very understanding and I didn’t feel intimidated at all, like I probably would with police.’ (TKSS Program User #1)*

One program user interview underscored the importance this non-judgmental and non-confrontational approach in reducing harms in nightlife spaces. Here, the program user described a potentially life-threatening situation where a friend was in-and-out of consciousness following heavy poly-drug use. While initially reluctant to contact an ambulance due to both his own drug intoxication, the intoxication of his friend, and a general fear of ‘getting in trouble with police’, conversations with the TKSS Ambassadors convinced both program users to seek emergency medical help. This finding is significant and speaks to the crucial HR role that TKSS plays in sites of high-level risk for AOD-related harm.

*‘We were freaking out a bit because I had drugs in my system and my friend was passing out and also had drugs in her system. I really didn’t know what to do. The people in the service really calmed me down and we eventually agreed that we both needed to go to hospital.’ (TKSS Program User #5)*

This non-judgement approach was key in de-escalating volatile and potentially harmful situations, reducing risk-prone behaviour and violent incidents and resulting in positive safety outcomes. Much of this positive effect was attributed to the approach and delivery of program services by TKSS staff and volunteers who were universally regarded by stakeholders as effective teams who have been central to the successes of the program in meeting its intended objectives. Team culture was highlighted as a key driver of positive interactions with the public, with many stakeholders regularly remarking on the ‘friendly’ and ‘inviting’ approach taken by staff even in the face of challenging or difficult situations.

*I think that is all down to their approach, they approach softly and people don’t see them as a threat…We see a lot of issues being resolved because they go about their work in that way’ (City Ranger #1)*

*‘They go in, they’re calm, they’re nurturing. Police sometimes have more of an authoritarian stance but these ones they appear to listen, comfort and engage.’ (NSW Police #1)*

Stakeholders also perceived the TKSS program as key to identifying and offering early intervention to individuals who may be at risk of harm. A&E and NSW Ambulance staff reported that the interventions by the TKSS program regularly resulted in a substantial reduction in the potential harm they may have experienced.

*‘If a patient is sent to the emergency department by the [TKSS] team when they call an ambulance, we will be receiving a patient that is intoxicated, drugged, call it whatever you want, but there is a chance that we would have received that patient later for doing something silly or being hit by a car or getting involved in a fight. So, that’s a main impact that we see, that we might see that patient intoxicated because it’s not safe to be in the street but that [major incident] didn’t happen. If no one called the ambulance, we would be seeing a different patient hours later.’ (A&E Staff Member #3)*

3. *Improving efficiency and effectiveness of emergency and other services operating in nightlife settings* Stakeholders regularly asserted that the TKSS program had been valuable in acting as an intermediary between service providers, often functioning as a critical triage service for serious incidents. The TKSS program was praised by emergency medical professionals and police for its ability to manage ‘at risk’ individuals and escalate the incident with emergency services as or if necessary. In providing this service, police, ambulance, and A&E staff reported that presence of the TKSS program allowed them to better manage their own resources by freeing them from the workload of dealing with high volumes of purely alcohol-intoxicated nightlife revellers. Program users and stakeholders noted that without the TKSS program, many more intoxicated revellers would end up in ‘risky’ and vulnerable situations, in hospital beds or in police stations simply because they didn’t have anywhere else to go for appropriate care:

*‘There is just no one out there who is really well equipped to deal with heavily intoxicated people. Like, we do it all the time, but are we really the best people to be doing this? Most of the time they end up with us simply because there is no one else to take care of them…The Take Kare program is providing options that are much more appropriate for the level of care needed in most cases’ (NSW Ambulance #4)*

*'It’s definitely assisted police I believe with taking some of the workload off the police who can be doing other things, dealing with other matters. For all police I think young persons are high care, no doubt about that, especially if they’re intoxicated, all that sort of thing, and it takes hours and hours and hours of police time to get that person home safely.' (NSW Police #4)*

The nature of the TKSS program and its interaction with other stakeholders in Sydney’s NTE led many interviewees to view the service as a ‘hub’, acting as a conduit between different agencies operating in nightlife settings. Stakeholders felt that the presence of the TKSS program had been significant in developing relationships between organisations that previously had minimal or less established levels of communication, creating a cooperative eco-system of service providers that better worked together to meet the needs of its users. Facilitating this coordination and collaboration with other service providers operating in Sydney’s key nightlife precincts was seen as a strength of the program and something that ultimately improved safety in the city at night.

*‘From the point of view of looking at better communication and coordination between stakeholders in the city at night. So, the program coming on board was an opportunity to go, "Okay. These guys are a conduit."... I mean, they're not coordinators but they're sort of a conduit to sort of bring it all together’ (City of Sydney Staff #1)*

*Look, we haven’t always had a great working relationship with the police, for many reasons and I think because of that we haven’t always wanted to contact the police, and some people don’t want to involve an ambulance if they are just drunk, or feeling off. What having the TKSS program has meant is that we have someone else to turn to, and when police come by, they see that we are working to get a good outcome for our clients, and I think they are less suspicious of us. (Licensee #3)*

The role of TKSS in linking together licensees, emergency services, the City of Sydney CCTV control room and other welfare services was seen as contributing to the efficiency and effectiveness with which each of these services function and fostered more productive working relationships between groups that have not always worked closely together:

*I think it is great that venues are directing people to the Safe Spaces. Like, before, venues use to just kick people out and hope for the best. This created really unsafe situations and we saw it go wrong a lot in Kings Cross. People would pass out, get caught in bad situations. There are a lot of people out there who prey on these people, and so having a space and service where people can go is a really good thing. (NSW Police #3)*

### Perceived weaknesses of the TKSS program

Stakeholders revealed several perceived weaknesses of the TKSS program including: (1) a lack of public awareness around the program; (2) the ability of the program to service its current localities given staff and volunteer levels; and (3) misunderstandings regarding the scope and function of the TKSS program by some stakeholders.

1. *A lack of public awareness around the program.* The most consistently identified weaknesses of the program across stakeholders related to a lack of public awareness of the program and its role. Here, interviewees regularly noted that most people out in nightlife precincts simply didn’t know that TKSS existed or, if they did, they weren’t sure what service it provided. Many program users, for example, said that they were not aware of the Program before their initial contact with the Ambassadors:

*‘I had no idea they existed, so I think it would be good if that was more known, just to give people that kind of sense of a bit of security. Like, I don’t want it to encourage people to get, you know, more drunk just because they know that someone’s there but I think it would be good to advertise there's like a safe space, even for people who aren’t drinking, so to speak.’ (TKSS Program User #8)*

*‘I guess the biggest question I would have is do enough people know about the program and what it does?...A program like Take Kare is probably limited in its impact because enough young people just don’t know about it… young people who need help still might not know it is there and that means they are not as effective as they might be if more people knew where they could go for help.’(NSW Ambulance #4)*

2. *The ability of the program to service its current localities given staff and volunteer levels.* It was asserted by stakeholders that the size of the locations the TKSS program services is a major challenge given the staff and volunteer resources available to the program, ultimately limiting the impact that the program can have. This was noted as critical given the proactive nature (and noted benefits detailed above) of the roving teams, with stakeholders stipulating that either an expansion of the program or a re-evaluation of its geographical reach was necessary. These operational issues were of particular concern to stakeholders working within the TKSS program.

*‘Operationally what could be improved? I think that it should be reconsidered around the footprint of the CBD site, what kind of area they're looking to cover. I think it's too big, in my opinion. Trying to survey a range from as far north as The Rocks, to as far south as Central Train Station, and to as far east as Taylor Square, and Oxford Street…it’s just too big’ (TKSS Staff #1)*

*‘I think again what's unique is the ambassadors sort of roving around the city. I guess it's something about not just waiting for people to come to you, but being really proactive and going out looking in the dark alleys where crimes might often happen. Or looking yeah, in the gutter or on a side street where someone might be vomiting or vulnerable. I think that's where a lot of our incidences really actually come from. Like if you stay in the one spot you don’t see that much, but when you are roving you do ... I think we've noticed it sometimes in King's Cross or in the city where we haven't had enough teams to rove in every direction.’ (TKSS Staff #3)*

3. *Misunderstandings regarding the scope and function of the TKSS program by some stakeholders* Some stakeholders and program users identified concerns regarding the sometimes-misplaced enthusiasm of TKSS staff and volunteers in situations that required more formal service responses. In some cases, it was perceived that TKSS staff had attended incidents where emergency health services should have been engaged immediately. Some stakeholders suggested that this issue may be the product of a misunderstanding of the role and scope of the TKSS program amongst other services and organisations, resulting in either the overuse or underuse of the program. While the limited authority the volunteers hold makes them more approachable to revellers, it also has the potential to place them at risk when referred to an incident they may not be equipped to handle. Likewise, it was indicated that some stakeholders may not have a complete understanding of the resources that the TKSS program has access to, and that this lack of knowledge has the potential to promote negative outcomes for users of the program.

*‘Maybe there's like a misunderstanding of our role by different stakeholders. Like I know we've been called in situations where there's like a first aid situation where really an ambulance should've been called straight away. And it's kind of ... Well that, five, 10 minutes between when you called and got there, like they could've been an ambulance’ (TKSS Staff #1)*

## Discussion

This study provides important qualitative insight into the strengths, weaknesses and stakeholder perspectives related to the operation of a safe space and roaming ambassador nightlife HR service that operated in Sydney between 2014 and 2022. The TKSS program aimed to improve the safety and amenity of the urban public domain by providing a HR service where vulnerable people in nightlife settings could access support and a safe place. Critically, this study both supports and extends the current evidence base around the efficacy and impact of peer-to-peer HR services deployed in nightlife settings. In particular, this study demonstrates the role that safe space programs can play in relation to supporting safety and wellbeing in the public domain and connecting-up other nightlife services critical to the delivery of a safe and vibrant night-time economy. Such service provision is particularly needed given the widespread use of liquor regulation around the “responsible” service of alcohol (itself widely considered a form of harm reduction policy) that requires licensed premises in many countries to cease service to and evict people showing obvious signs of intoxication. In fact, international research has shown that some the most severe incidents of physical harm can result from these eviction practices and subsequent patron conflict outside venues [2, 7]. Findings from this study highlight that having a service dedicated to the delivery of care to vulnerable nightlife patrons can encourage venue security and staff involved in enacting eviction to involve safe space staff in the process, ensuring they are not left in situations of risk and, as one participant described it, providing “a safety net” in the city after dark. The volume of public interventions made by the TKSS program since its inception in 2014 speaks to how frequently nightlife revellers require this type of support and care following time in, or ejection from, licensed venues in inner Sydney and demonstrates the need for reform in the service of alcohol and provision of care in these settings.

This study also highlights the important role of roving HR services in providing triage and mitigation against more intensive intervention for intoxicated nightlife revellers. It is widely and routinely reported that night-time AOD use and attending harms are associated with significant costs to individuals, businesses, and the state through provision of health care, policing services, and other impacts on the economy [75–78]. Findings from this study highlight that the operation of the TKSS program was an effective method for reducing contact and de-escalating conflict between intoxicated revellers and police, interactions that often result in unnecessary antagonism and criminalisation. These negative interactions were perceived by participants of this study as much less likely to occur following TKSS interventions. Here, findings mirror that of previous research around help-seeking in the context of nightlife and music festivals which highlight the key role that peer-to-peer services can play in acting as a triage between key services that are often needed in situations of AOD intoxication, distress or following altercations in these settings [27, 39]. Also replicating previous findings, peer-based roving ambassadors working for TKSS were seen by program users as far less intimidating and judgemental than police and therefore less likely to pose a threat to someone in need of help [27, 39]. Frontline services personnel interviewed also cited how much more appropriate a care-based intervention was for revellers who were nothing more than intoxicated in a public place. Historically in NSW, people who were heavily intoxicated or passed-out in public spaces would often end up in A&E or in police custody [79], neither of which are an appropriate use of resources, nor the best result for those involved. This outcome follows similar findings from studies which have demonstrated the unmet need serviced by programs like TKSS, and the important and more appropriate alternative that such services provide in the delivery of interventions when compared to traditional emergency services [80].

Providing an alternative to police in the regulation and de-escalation of conflict in nightlife settings not only has the direct benefit of preventing the criminalisation of revellers, but also in reducing injury. The ability of TKSS ambassadors to identify vulnerable situations and people and provide early intervention minimised the potential for future harm and was frequently identified as the greatest value-generating activity of the program. Drug and alcohol affected program users felt comfortable and willing to divulge information to TKSS ambassadors they wouldn’t readily tell other service providers. The provision of non-judgemental support assisted uptake of the service, enhanced the ability to deliver important messages about risk reduction, and, in doing so, allowed action and relations that minimised harm. Again, these findings mirror those of other research that have highlighted how drug-affected young people often actively avoid police and medical services for of fear of judgement or arrest, or engage in more harmful consumption practices to avoid contact/detection by them [27, 39]. The avoidance of such services in instances of heavy intoxication can be fatal, and so again demonstrated the critical role of TKSS in getting program users the help they need.

The role of the TKSS program in increasing communication and cooperation between service providers was a clearly stated strength that allowed key service providers to conduct their work more efficiently. Ensuring a safe night-time economy requires the collaboration of a range of sectors to provide a co-ordinated approach to management [1, 2, 24, 81–84]. International research has identified the strong need for co-ordination and effective partnership in developing and managing sustainable, healthy, and safe night-time economies [81–84], including the need to move away from reactive enforcement towards a more upstream approach to incorporating proactive prevention, intervention and a public health approach to policing [83]. This data further supports the strong need for a co-ordinated approach to the provision of services in such environments.

## Limitations

This study was completed in Sydney, Australia, during a period of highly restrictive regulations governing nightlife and the service of alcohol in licensed premises in the areas in which the TKSS program was operating. While it cannot be fully known how this regulatory context may have impacted the data collected, there is no doubt that engagement in Sydney nightlife was significantly impacted by the “lockout laws” and so issues reported may have been influenced by this operational context, and may therefore limit the transferability of these findings to other locations. Further, recruitment of program users was difficult and slow given that many who used the service during data collection were heavily intoxicated and unable to engage with advertising materials. This issue undoubtedly impacted on the final number of program users who took part in interviews, but the level of data saturation achieved with only this relatively small number of participants and the consistency with findings from the survey (reported elsewhere) indicated that it was sufficient to be considered a good reflection of program user sentiment regarding the TKSS service.

## Conclusion

This study demonstrates the complex relationships that exist around the delivery of harm reduction in nightlife settings. In particular, it highlights the relative lack of servicing of public nightlife settings and the value of safe spaces/ peer-to-peer safety ambassador programs in linking up care and filling this service gap. It documents the extended benefit across key stakeholder groups for the delivery of proactive and non-judgemental harm reduction and, in doing so, provides critical evidence around their efficacy in reducing AOD-related harms in the night-time economy.

## Data Availability

The datasets generated and/or analysed during the current study are not publicly available, but are available from the corresponding author on reasonable request.
